# Methylation Landscapes of Cartilage in Hip Osteoarthritis

**DOI:** 10.1155/genr/5540232

**Published:** 2026-01-05

**Authors:** Ruiyang Jiang, Maochun Wang, Guihua Tan, Jie Lv, Xiaoyu Jin, Yuan Liu, Rui Wu, Dongquan Shi

**Affiliations:** ^1^ Division of Sports Medicine and Adult Reconstructive Surgery, Department of Orthopedic Surgery, Nanjing Drum Tower Hospital Clinical College of Xuzhou Medical University, 321 Zhongshan Road, Nanjing, 210008, Jiangsu, China; ^2^ Department of Plastic Surgery, The Affiliated Friendship Plastic Surgery Hospital of Nanjing Medical University, Nanjing, Jiangsu, China; ^3^ Division of Sports Medicine and Adult Reconstructive Surgery, Department of Orthopedic Surgery, Nanjing Drum Tower Hospital, The Affiliated Hospital of Nanjing University Medical School, Nanjing, China, nju.edu.cn; ^4^ Division of Sports Medicine and Adult Reconstructive Surgery, Department of Orthopedic Surgery, Nanjing Drum Tower Hospital Clinical College of Traditional Chinese and Western Medicine, Nanjing, China

**Keywords:** AMPK, cartilage, genome-wide DNA methylation, hip osteoarthritis, PI3K-AKT

## Abstract

**Objective:**

To elucidate different methylation landscapes between cartilage of femoral neck fracture and preserved and damaged cartilages in hip osteoarthritis (OA).

**Methods:**

Genome‐wide DNA methylation data were acquired from two data sets in GEO database (GSE63106 and GSE63695), which were based on Illumina HumanMethylation450 BeadChip arrays. A total of 63 hip samples were selected for further analysis, including 19 cartilages obtained from patients with femoral neck fracture, 14 preserved cartilages, and 30 damaged cartilages obtained from patients with OA. We identified the differential methylated positions (DMPs) and genes between different cartilage groups.

**Results:**

There were 116,750 DMPs and 51,200 DMPs identified in preserved and damaged cartilages compared to cartilage in femoral neck fracture, respectively, while there were no signals found between preserved and damaged cartilages. Gene ontology analysis showed that most of differential methylated genes were enriched in extracellular matrix and structure organization, collagen‐containing extracellular matrix, and KEGG enrichment highlighted PI3K‐AKT and AMPK signaling pathways, which were known to be crucial for the progression of OA. Further construction of protein–protein interaction networks with differential methylated genes elucidated molecular basis of the disease. Three hypermethylated genes (NOTCH1, GREM1, and DYSF) and three hypomethylated genes (HDAC4, S100A10, and RUNX1) were selected to detect the relative expression in different cartilages, and their expression was correlated with the methylation status within the genes.

**Conclusion:**

We demonstrated the differential methylated genes across the whole genome not only on preserved cartilage but also on damaged cartilage during OA. The molecular network highlighted the potential therapy targets which may be involved in the initiation or progression of the disease.

## 1. Introduction

Osteoarthritis (OA) is a common degenerative joint disease, which is characterized by destruction of articular cartilage and accompanied by changes in other joint tissues [1–3]. It is widely known that a lot of risk factors are responsible for OA, including aging, gender, obesity, genetics, and epigenetics [4, 5]. Genetic approaches using genome‐wide association studies (GWAS) have identified lots of loci and genes associated with OA [6–8], while the epigenome of OA is not fully elucidated.

Recently, GWAS has identified TGFB1, FGF18, CTSK, and IL11 to be the likely effector genes that have therapeutics approved or in clinical trials for OA, using UK Biobank data [6]. A genetic study of 9 populations including 826,690 individuals has identified 100 risk variants of 11 OA‐associated phenotypes, 52 of which are newly found [7]. The most common targets of OA discovered so far are RUNX2, TGFB1, GDF5, COLGALT2, PLEC, MGP, ALDH1A2, and RWDD2B [9]. Although lots of associated variants have been discovered by GWAS, most of the genes do not show any significant change at RNA or protein level.

DNA methylation is associated with the regulation of gene transcription, most of which are generally involved in repression of gene expression, and this is one of the most studied epigenetic mechanisms in OA [10]. Several studies have shown that DNA methylation has regulatory functions in the pathogenesis of OA using human samples and mouse models [11, 12]. Methylation of gene promoters or enhancers, like the 1500 nt regions upstream transcription start site (TSS1500), was known to inhibit gene expression, while most of the methylation within gene body was correlated with upregulation of gene expression [13]. Genome‐wide approaches have revealed methylation alterations in many genes which are involved in OA, including extracellular matrix (ECM)–associated genes COL2A1 and MMP13, gene members of TGFβ signaling pathway TGFB1, SMAD2, SMAD3, and BMP6 [14, 15], microRNAs, circular RNAs, and long noncoding RNAs [5, 16]. Although large amounts of epigenome data have revealed DNA methylation changes in OA, it is still not explicit whether these changes are crucial or consequential for the progression of the disease.

Here, we used genome‐wide DNA methylation data of hip cartilage of femoral neck fracture and preserved and damaged hip cartilages of OA to identify differential methylated positions (DMPs) and genes, which revealed enrichment of biological process and signal pathway involved in the pathological process of OA. Further construction of molecular networks was helpful to discover the gene molecular interaction of OA at methylation level. As far as we knew, there was currently no methylation analysis combining these three different states of cartilage, and our methylation analysis of femoral neck fracture and preserved and damaged hip cartilages might help with early diagnosis of OA.

## 2. Methods

### 2.1. DNA Methylation Data Sources

Genome‐wide DNA methylation data were acquired from Gene Expression Omnibus (GEO) database, and the access numbers were GSE63106 and GSE63695 [14, 17, 18]. Both data sets were based on Illumina Infinium HumanMethylation450 BeadChip arrays, which contained probes of 484,412 CpG sites across the whole genome, covering almost all the reference genes [19]. All hip cartilages were selected, including 19 negative control cartilages (NCs) from patients with femoral neck fracture and 16 damaged cartilages from patients with OA in GSE63106 (NC = 19, OA = 16) and 14 preserved cartilages (PRs) and 14 damaged cartilages obtained from patients with OA in GSE63695 (PR = 14, OA = 14). The characteristic of all patients is shown in Supporting Table 1. The raw data were merged by probe names, and a total of 63 hip cartilages and *β* values of 351,512 unique probes were screened for further analysis. Data quality control was performed by ChAMP (v2.34.0), minfi (v1.50.0), and wateRmelon (v2.10.0) with R packages (Supporting Figures 1A and 1B).

### 2.2. Identification of DMPs and Gene Enrichment

DMPs were identified by champ.DMP function in ChAMP R package. There were 116,750 DMPs found in NC vs. PR and 51,200 DMPs found in NC vs. OA, while there was no difference between PR and OA. Volcano plot was constructed by ggpubr (v0.6.0) R package, and hypermethylated and hypomethylated positions were filtered by the absolute value of deltaBeta over 0.2 and *p* value less than 0.05. Heatmaps of all DMPs in NC, PR, and OA were plotted by pheatmap (v1.0.12) R package (Supporting Figures 1C and 1D). Differential methylated genes were derived after the annotation of hypermethylated and hypomethylated positions, and the enrichment of Gene Ontology (GO) and Kyoto Encyclopedia of Genes and Genomes (KEGG) was analyzed by clusterProfiler (v4.12.0) R package. Venn diagram of differential methylated genes in NC vs. PR and NC vs. OA was created by VennDiagram (v1.7.3) R package. Differential methylated genes overlapped in both comparisons were imported in STRING database and constructed protein–protein interaction networks, which were further edited with Cytoscape (v3.9.1). *β* values of three hypermethylated genes (neurogenic locus notch homolog protein 1 [NOTCH1], Gremlin 1 [GREM1], and dysferlin [DYSF]) and three hypomethylated genes (histone deacetylase 4 [HDAC4], S100 calcium‐binding protein A10 [S100A10], and Runt‐related transcription factor 1 [RUNX1]) were extracted by clusterProfiler R package, which was shown as mean ± SD by GraphPad Prism 9 (v9.5.1). Statistical significance in different groups was calculated by analysis of variance (ANOVA) method.

### 2.3. Clinical Specimen

The study protocol received approval from the Ethics Committee of Nanjing Drum Tower Hospital, Affiliated Hospital of Nanjing University Medical School (2022‐176‐01). Human hip cartilages were collected from six patients of femoral neck fracture and six patients with hip OA who underwent total hip arthroplasty. The cartilage in the relatively intact area of the hip joint of patients with femoral neck fractures serves as the NC. The hip OA cartilage samples were divided into preserved and damaged areas, the intact cartilage on the surface is classified as PR, whereas the damaged regions are designated as OA cartilage (OA).

### 2.4. Quantitative Real‐Time Polymerase Chain Reaction (qPCR)

Total RNA was extracted using RNA‐Quick Purification Kit (ES Science, China), and cDNA was synthesized with HiScript III RT SuperMix for qPCR Kit (Vazyme, China) following manufacturer’s instructions. qPCR amplification of diluted cDNA was used with ChamQ Universal SYBR qPCR Master Mix (Vazyme, China), which applied on LightCycler 480 PCR system (Roche, Switzerland). Relative gene expressions were calculated with the 2^−ΔΔCt^ method, using GAPDH as internal control. Primer sequences are provided in Supporting Table 4.

### 2.5. Protein Extraction and Western Blot

Total proteins were extracted from chondrocytes using RIPA (#R0010, Solarbio) lysate buffer containing a 1 mM phosphatase inhibitor mixture (#B15002, Bimake, USA) and 1 mM benzoyl fluoride (#329‐98‐6, Solarbio). The protein concentration of the lysate was determined by the Bradford assay (#A55866, Thermo Scientific). Proteins were separated on a 10% sodium dodecyl sulfate‐polyacrylamide gel electrophoresis (#PG112, EpiZyme, Shanghai, China) and transferred to a polyvinylidene fluoride membrane (#IPVH00010, Millipore, USA) following standard procedures. After blocking the membrane with 5% milk (#1172GR500, Biofroxx) for one hour at room temperature, it was stained with NOTCH1 (#A19090, ABclonal), GREM1 (#A11595, ABclonal), DYSF (#A19572, ABclonal), HDAC4 (#A13510, ABclonal), S100A10 (#A13614, ABclonal), RUNX1 (#A2055, ABclonal), and *β*‐actin antibody (#4970S, Cell Signaling Technology). Horseradish peroxidase‐conjugated goat antirabbit/mouse IgGs (1:5000 #BL003A or #BL001A, Biosharp) were used as secondary antibodies. All signals were detected using the ChemiDoc XRS + Imaging System (Tanon, Shanghai, China). Quantitative analysis of protein density was performed using Image J (version 1.8.0).

## 3. Results

### 3.1. Quality Control of DNA Methylation Data

Genome‐wide DNA methylation data were acquired from GEO database, including two independent data sets (GSE63106 and GSE63695). All the hip samples were selected for further analysis, containing 19 NCs (NC = 19) obtained from patients with femoral neck fracture and 14 PRs (PR = 14) and 30 damaged cartilages (OA = 30) obtained from patients with OA. Principal components analysis (PCA) showed that NC and PR clustered separately, while OA group was not separated from other groups, which demonstrated the heterogeneity of OA (Figure 1(a)). Multiple dimensional scaling (MDS) plot of the 1000 most variable positions displayed difference with NC from femoral neck fracture and PR and OA from OA (Figure 1(b)). All the data were in good condition as shown in *β* density plot (Figure 1(c)), which indicated that the data resources were reliable. Correlation of all the data in different samples revealed that most of NC groups were clustered after normalization (Figure 1(d)).

Figure 1Data quality control of genome‐wide DNA methylation data in hip cartilage. (a) Principal components analysis (PCA) of all hip cartilages. Red circle, NC; blue square, preserved cartilage; green triangle, damaged cartilage. (b) Multiple dimensional scaling (MDS) plot of the 1000 most variable positions in all samples. Green circle, NC; purple circle, preserved cartilage; orange circle, damaged cartilage. (c) Density bean plots of methylation *β* values across the DNA methylation data. Green, NC; purple, preserved cartilage; orange, damaged cartilage. (d) Correlation of all the data between different samples. The depth of the color represents the strength of correlation between different samples.

**Figure:**
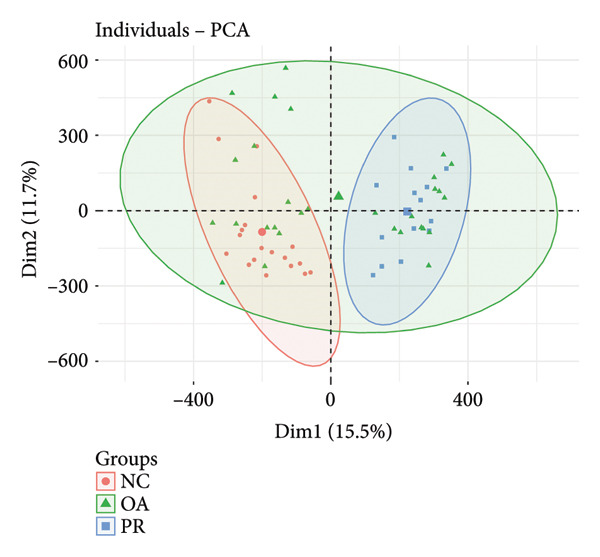
(a)

**Figure:**
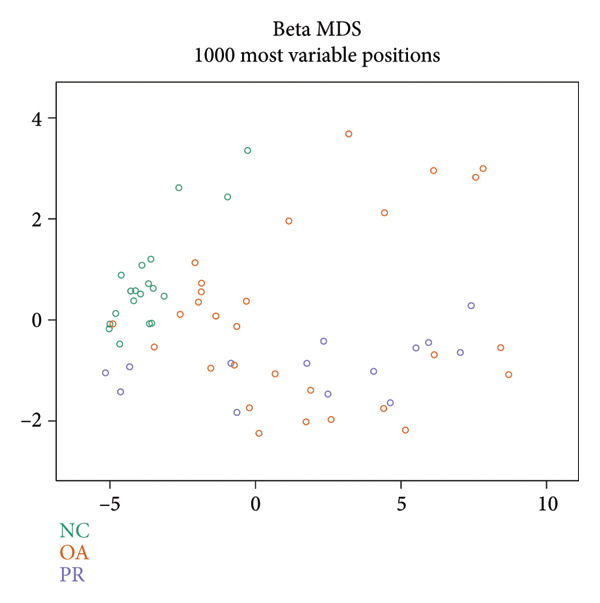
(b)

**Figure:**
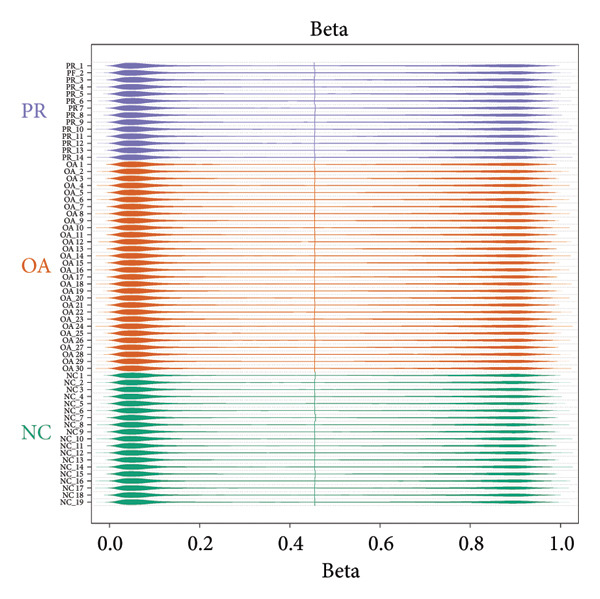
(c)

**Figure:**
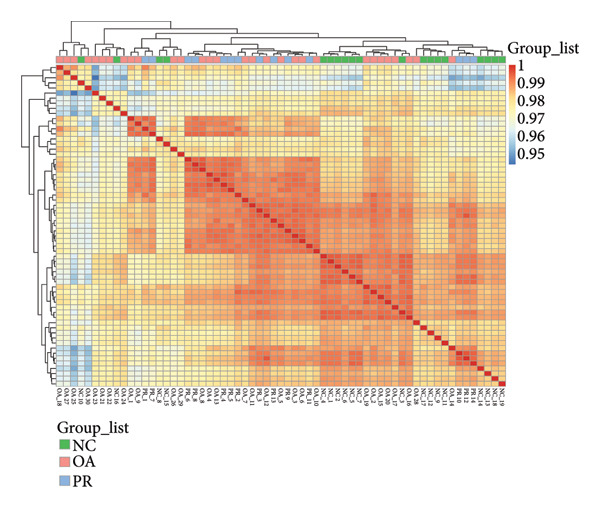
(d)

### 3.2. DMPs in NC, PR, and OA Cartilages

There were 116,750 DMPs in NC vs. PR, including 408 hypermethylated positions, 859 hypomethylated positions, and 115,483 stable positions (Figure 2(a), Supporting Table 2). There were 51,200 DMPs in NC vs. OA, including 216 hypermethylated positions, 441 hypomethylated positions, and 50,543 stable positions (Figure 2(b), Supporting Table 2). The results suggested more hypomethylated positions during OA. Although there were number differences between preserved and damaged cartilages, their proportion of distribution across the genome was relatively similar, which were uniformly distributed across the genome and mainly located in gene body and intergenic regions (Figures 2(c) and 2(d)). However, no methylated positions were found between PR and OA, which was inconsistent with the original data reports [18]. This might be due to our stricter screening criteria and more OA cartilage samples. Heatmap of top 20 methylated genes in NC vs. PR and NC vs. OA displayed different methylated conditions in femoral neck fracture and OA samples (Figures 2(e) and 2(f)). There were several genes which had different methylated positions, like ASAP2 (cg23902076, cg00013804), TNXB (cg12694372, cg15265085, cg24882324), DYSF (cg14196395, cg12245706), FGFR2 (cg10314760, cg20277356), and RUNX1 (cg04915566, cg01519261). Interestingly, there were more common hypomethylated genes in PR and OA, while fewer identical hypermethylated genes were found in PR and OA, which indicated similar and different methylation status in preserved and damaged cartilages.

Figure 2Volcano plot of differential methylated positions (DMPs) and heatmap of differential methylated genes in different samples. (a) Volcano plot of DMPs in preserved cartilage compared to NC. The most significant 6 DMPs were marked. (b) Volcano plot of DMPs in damaged cartilage compared to NC. The most significant 6 DMPs were labeled. (c) Feature proportion of hypermethylated and hypomethylated positions in NC and PR. (d) Feature proportion of hypermethylated and hypomethylated positions in NC and OA. UTR, untranslated region; IGR, intergenic region; TSS, transcription start sites. (e) Heatmap of top hypermethylated and hypomethylated genes in preserved cartilage and NC samples. (f) Heatmap of top hypermethylated and hypomethylated genes in damaged cartilage and NC samples. NC, negative control cartilage; PR, preserved cartilage; OA, damaged cartilage.

**Figure:**
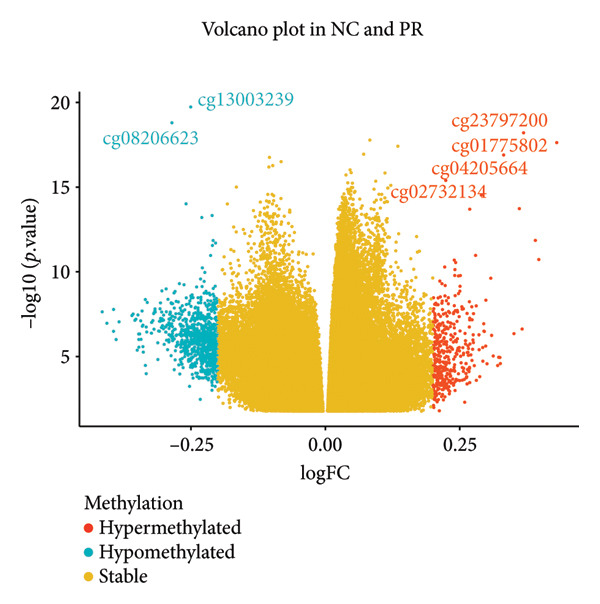
(a)

**Figure:**
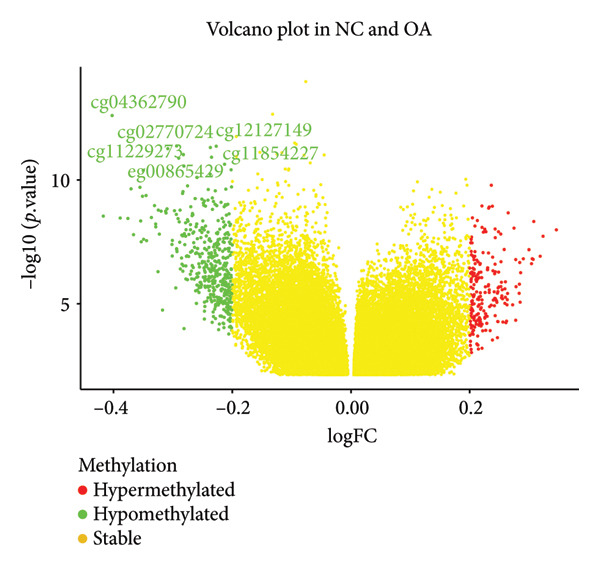
(b)

**Figure:**
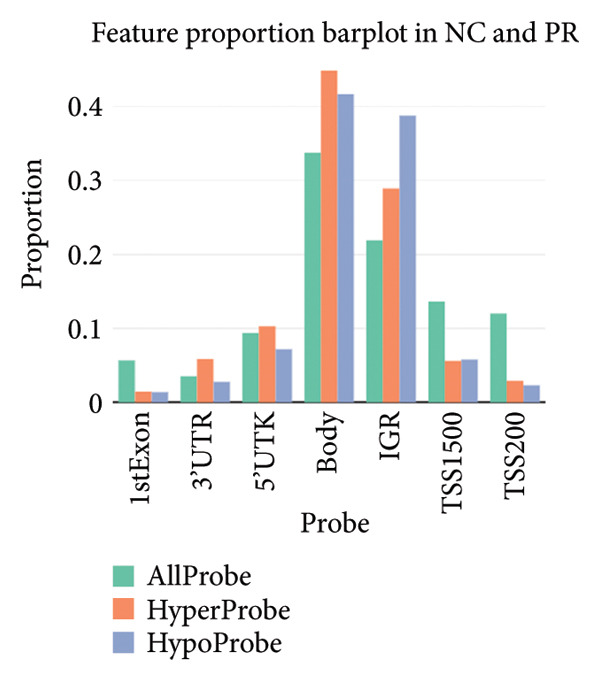
(c)

**Figure:**
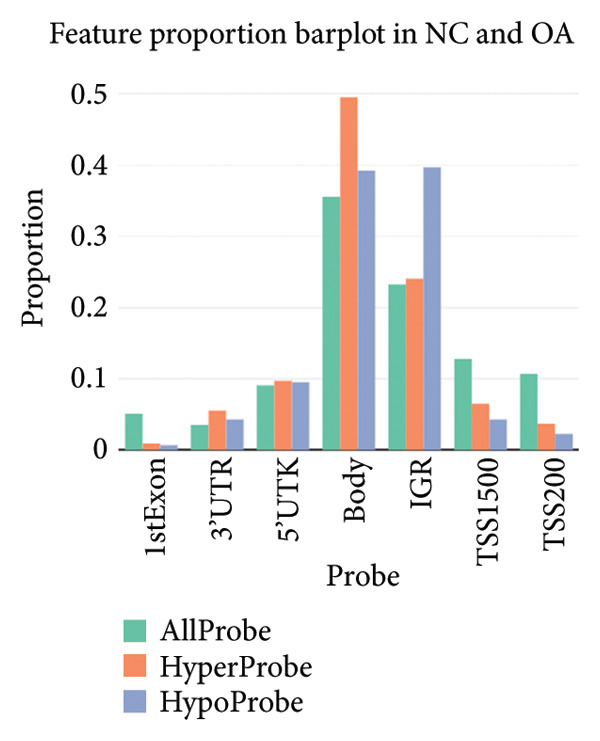
(d)

**Figure:**
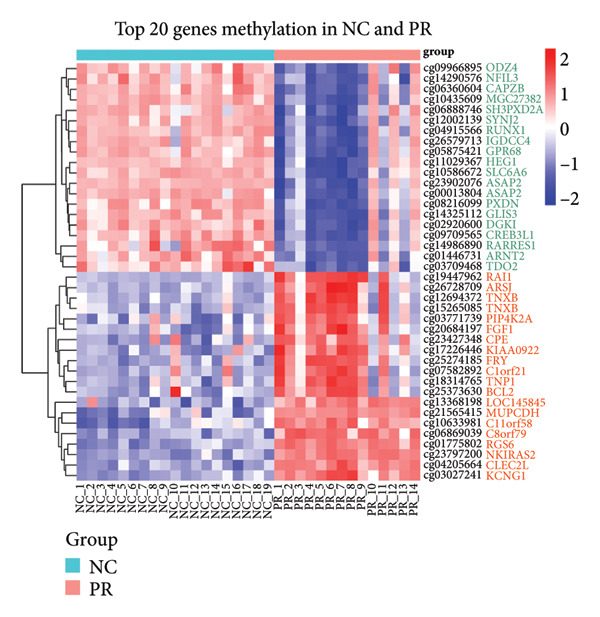
(e)

**Figure:**
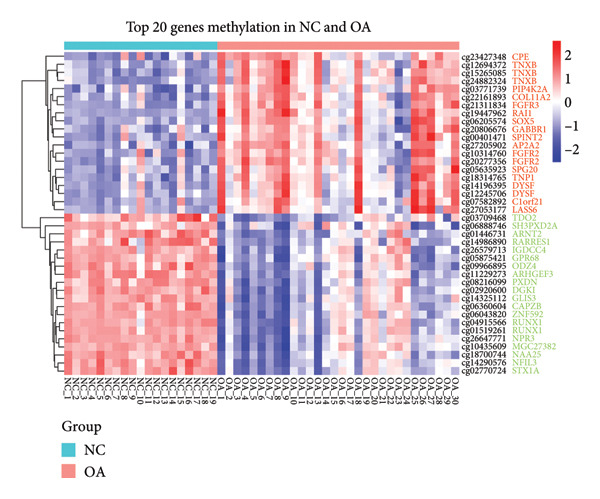
(f)

### 3.3. Gene Enrichment and KEGG Analysis in NC, PR, and OA

After annotation, 720 differential methylated genes were identified in NC vs. PR, containing 254 hypermethylated genes and 466 hypomethylated genes. There were 377 differential methylated genes in NC vs. OA, including 144 hypermethylated genes and 233 hypomethylated genes. Gene enrichment showed that most of the differential methylated genes were related to ECM and structure organization, collagen‐containing ECM (Figures 3(a) and 3(b), Supporting Table 3), which was consistent with the change of ECM in cartilage during OA. KEGG analysis in NC, PR, and OA revealed differential methylated genes mainly enriched in PI3K‐AKT signaling pathway and AMPK signaling pathway (Figures 3(c) and 3(d), Supporting Table 3), of which PI3K‐AKT signaling pathway was essential for chondrocyte survival and apoptosis and also involved in the development of OA.

Figure 3Gene enrichment of differential methylated genes. Gene Ontology (GO) of differential methylated genes in preserved (a) and damaged cartilages (b) compared to NC. Kyoto Encyclopedia of Genes and Genomes (KEGG) of differential methylated genes in preserved (c) and damaged cartilages (d) compared to NC. Red and blue colors represent the magnitude of the p.adjust values. The size of the circle represents the number of genes.

**Figure:**
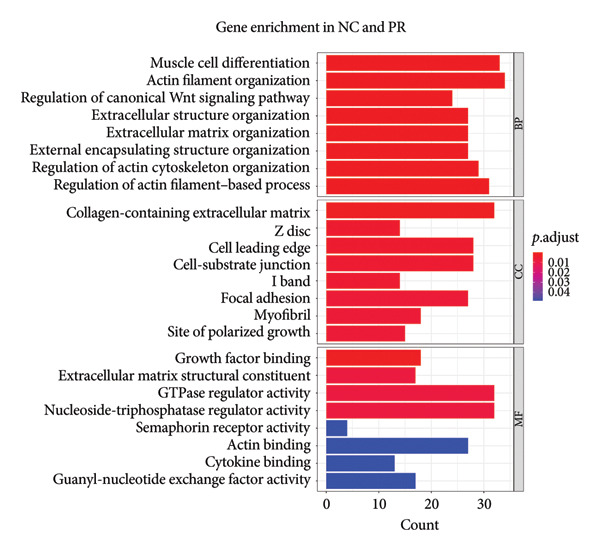
(a)

**Figure:**
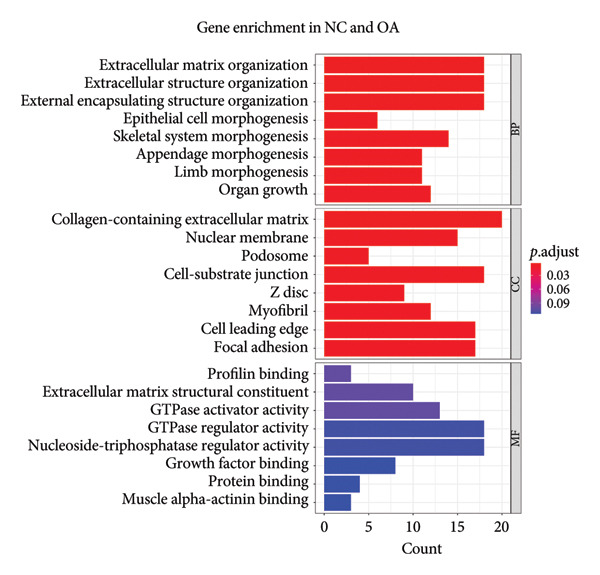
(b)

**Figure:**
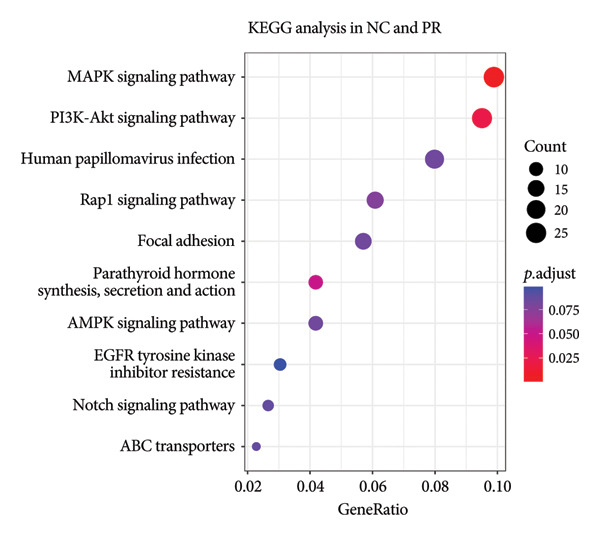
(c)

**Figure:**
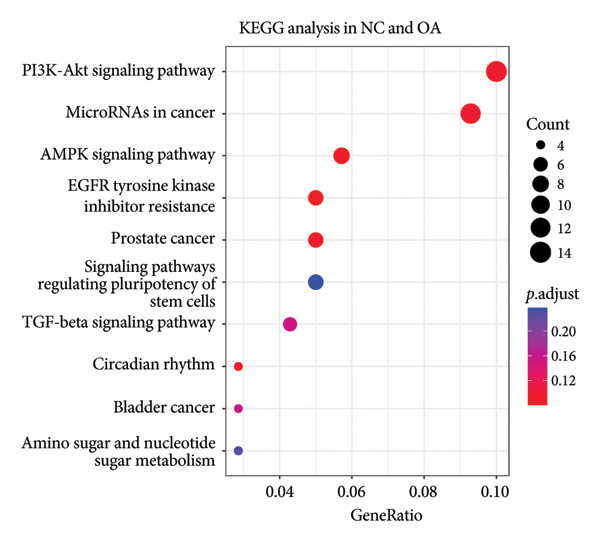
(d)

### 3.4. Hypermethylated and Hypomethylated Genes Verified in Hip OA

There were 308 differential methylated genes overlapped between NC vs. PR and NC vs. OA (Figure 4(a)). These genes were imported into STRING database and constructed molecular networks that might reveal the molecular mechanism of methylation in OA (Figure 4(b)). We chose six methylated genes that were overlapped in both groups and showed statistical significance in methylation to evaluate their expression in hip OA cartilage. Three identical hypermethylated genes, including NOTCH1, GREM1, and DYSF, exhibited significant hypermethylation in PR and OA, while three identical hypomethylated genes, including HDAC4, S100A10, and RUNX1, exhibited significant hypomethylation in PRs and OA (Figures 4(c), 4(d), 4(e), 4(f), 4(g), and 4(h)). Methylation of gene promoters or enhancers, like the 1500 nt regions upstream transcription start site (TSS1500), was known to inhibit gene expression, while most of the methylation within gene body was correlated with upregulation of gene expression [13].

Figure 4Molecular network of methylated genes and verification of six methylated genes. (a) Venn diagram of methylated genes between preserved and damaged cartilages compared to NC. (b) Protein–protein interactions of overlapped methylated genes in (a). (c–e) *β* values of hypermethylated genes NOTCH1, GREM1, and DYSF in NC and preserved and damaged cartilages. (f–h) *β* values of hypomethylated genes HDAC4, S100A10, and RUNX1 in NC and preserved and damaged cartilages. Data are shown by boxplot. (i–o) Western blot analysis and quantitation of indicated proteins in NC and preserved and damaged cartilages. (p–r) Relative mRNA expression of hypermethylated genes NOTCH1, GREM1, and DYSF in NC and preserved and damaged cartilages. (s–u) Relative mRNA expression of hypomethylated genes HDAC4, S100A10, and RUNX1 in NC and preserved and damaged cartilages. ^∗^Statistical significance. ^∗^ *p* < 0.05. ^∗∗^ *p* < 0.01. ^∗∗∗^ *p* < 0.001. ^∗∗∗∗^ *p* < 0.0001. ns represents there is no statistical significance. NC, negative control cartilage; PR, preserved cartilage; OA, damaged cartilage.

**Figure:**
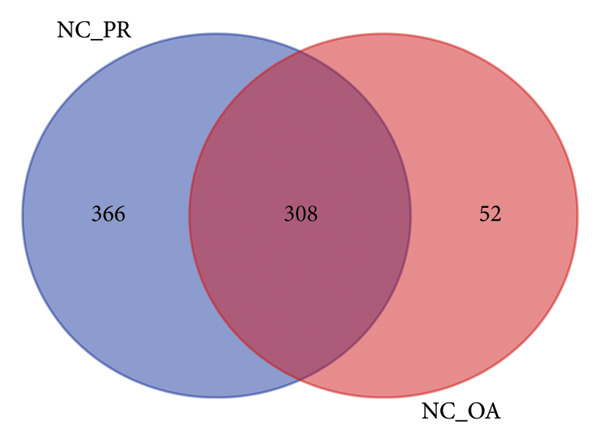
(a)

**Figure:**
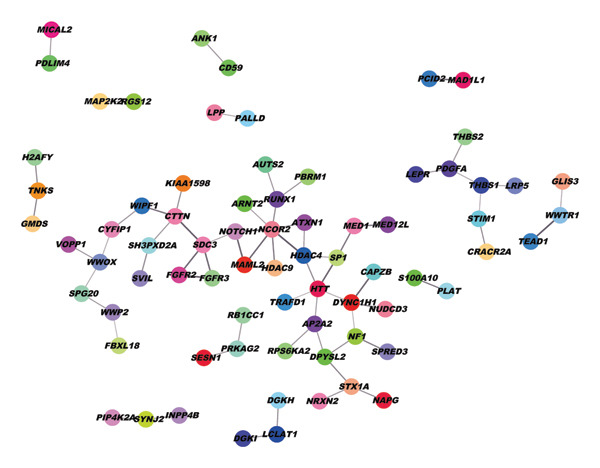
(b)

**Figure:**
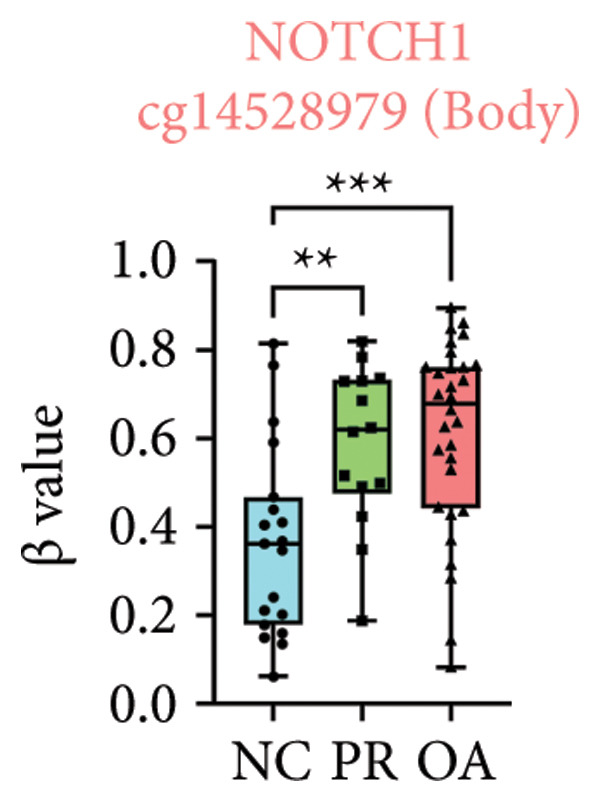
(c)

**Figure:**
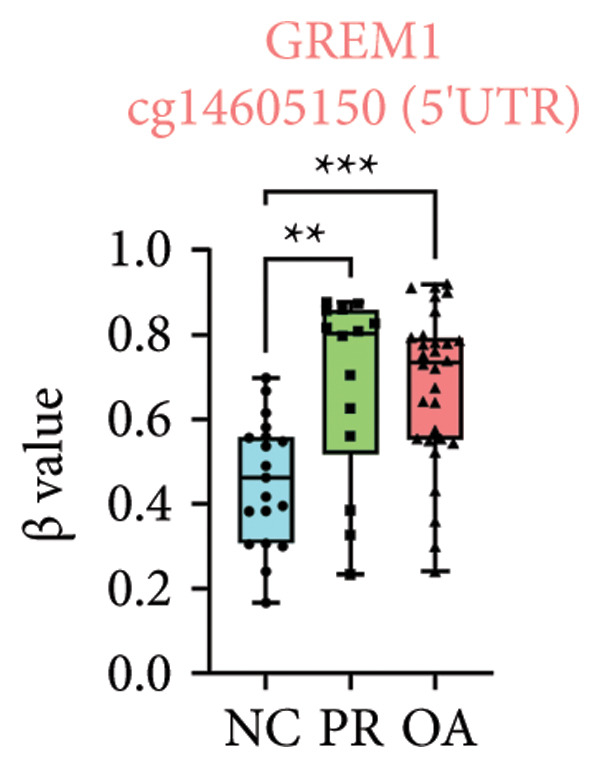
(d)

**Figure:**
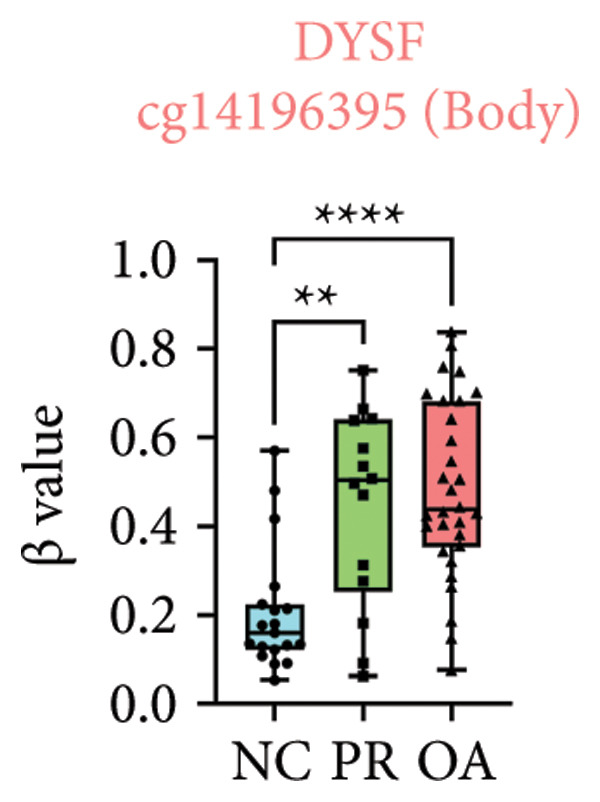
(e)

**Figure:**
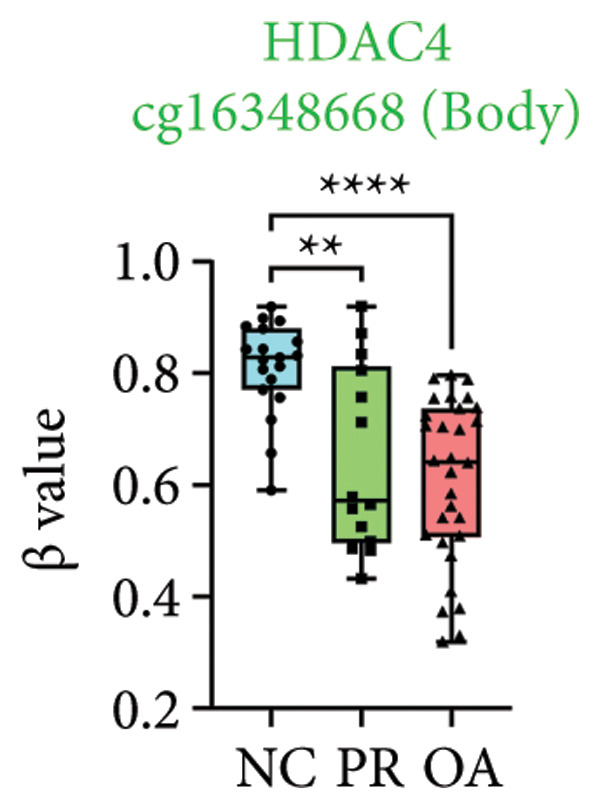
(f)

**Figure:**
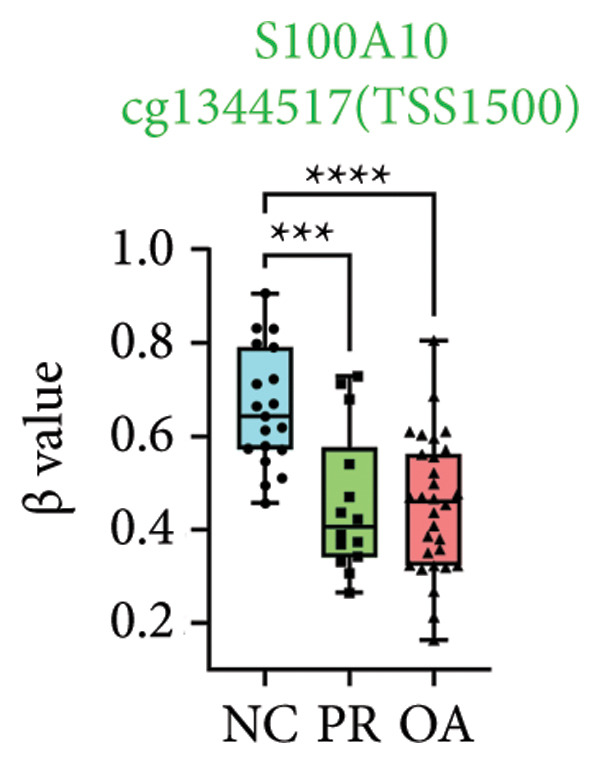
(g)

**Figure:**
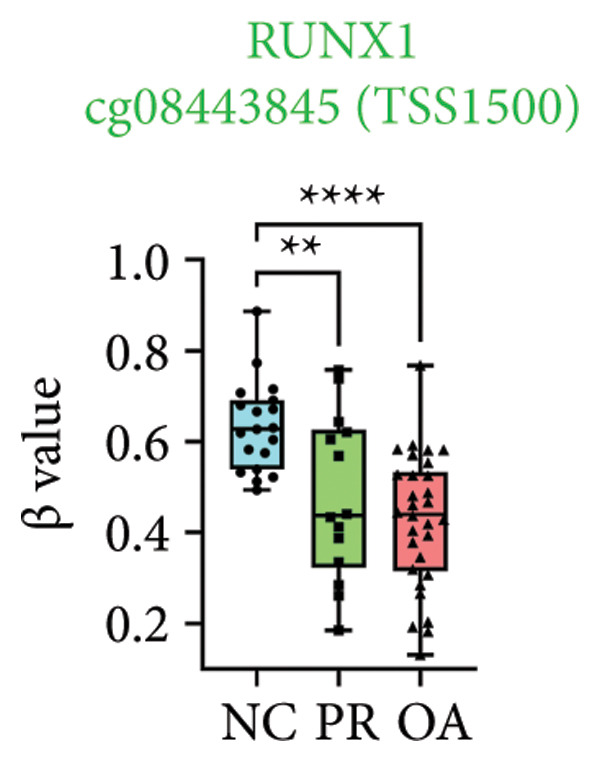
(h)

**Figure:**
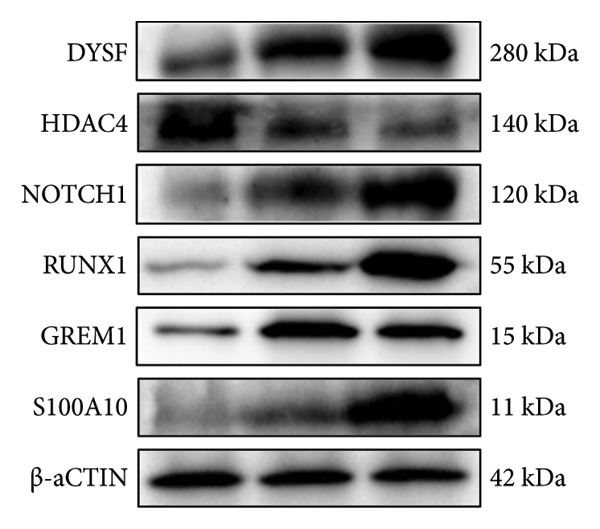
(i)

**Figure:**
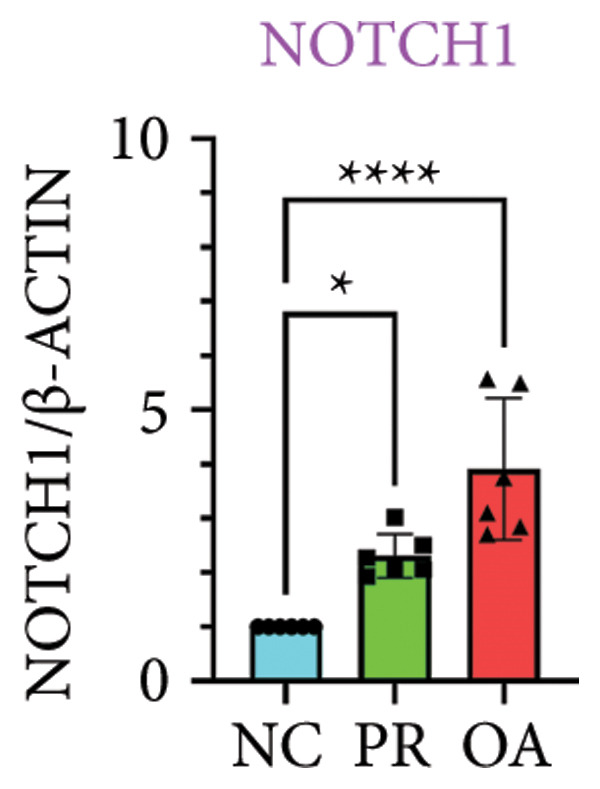
(j)

**Figure:**
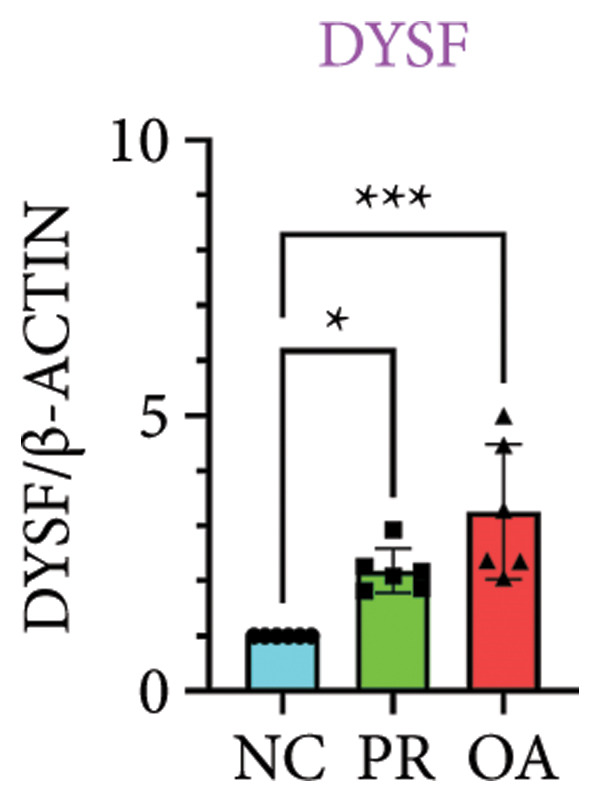
(k)

**Figure:**
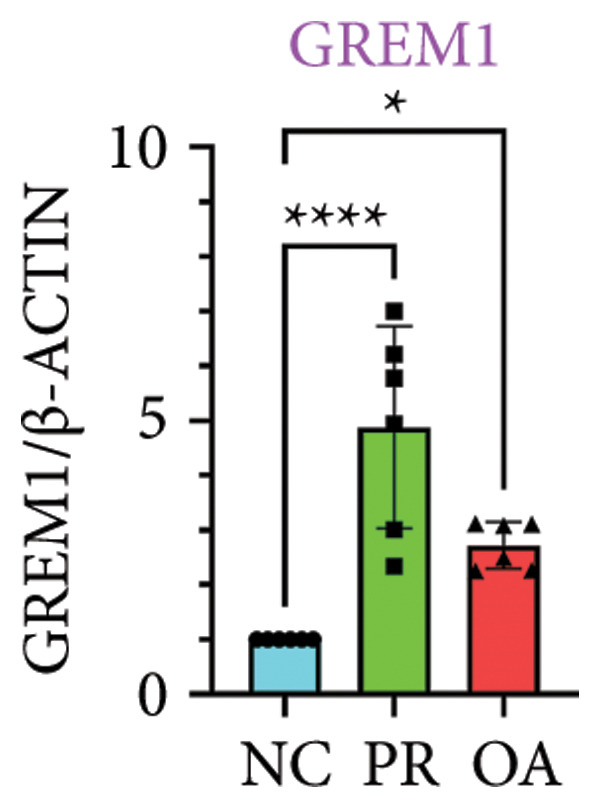
(l)

**Figure:**
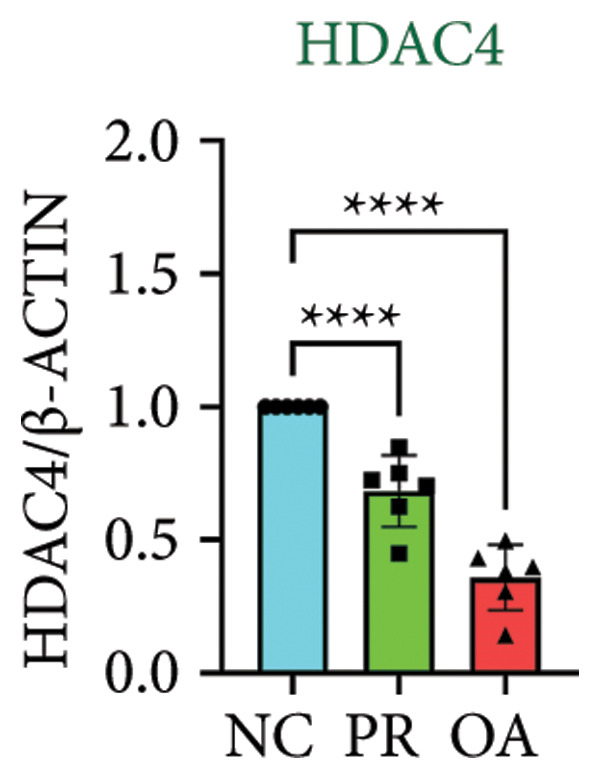
(m)

**Figure:**
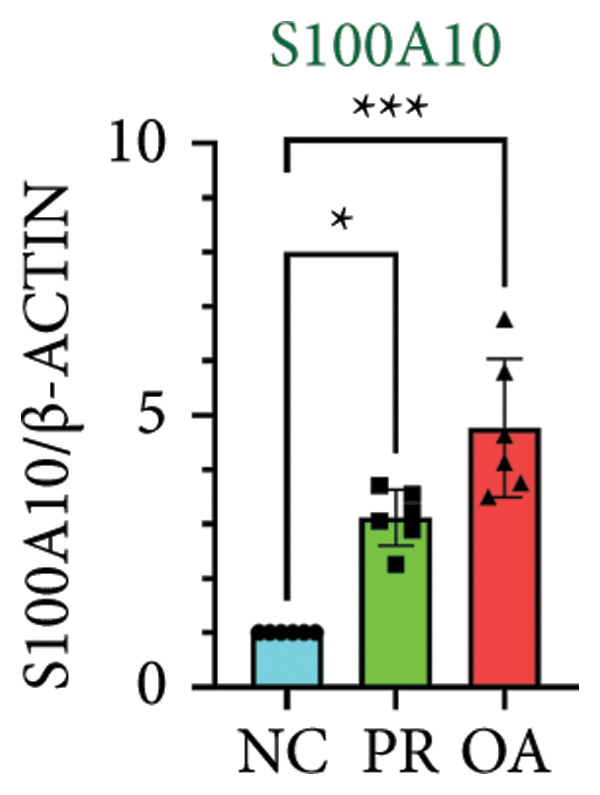
(n)

**Figure:**
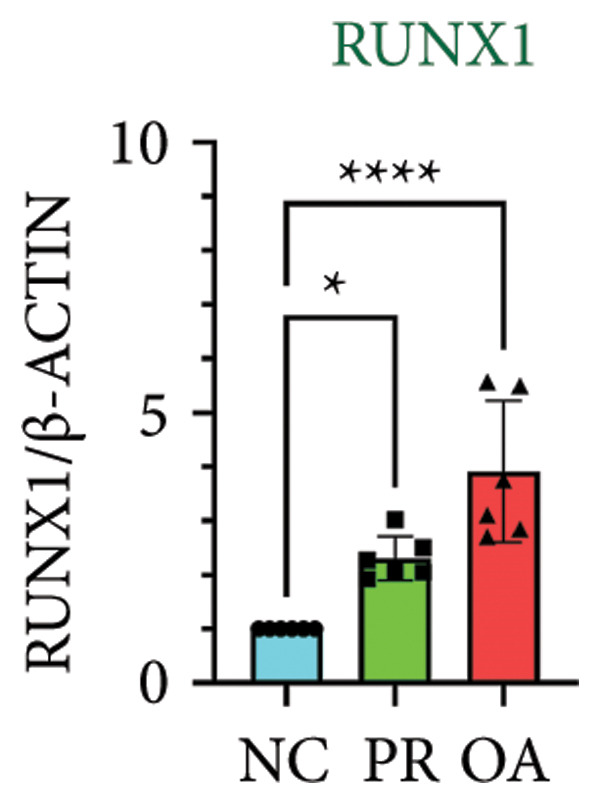
(o)

**Figure:**
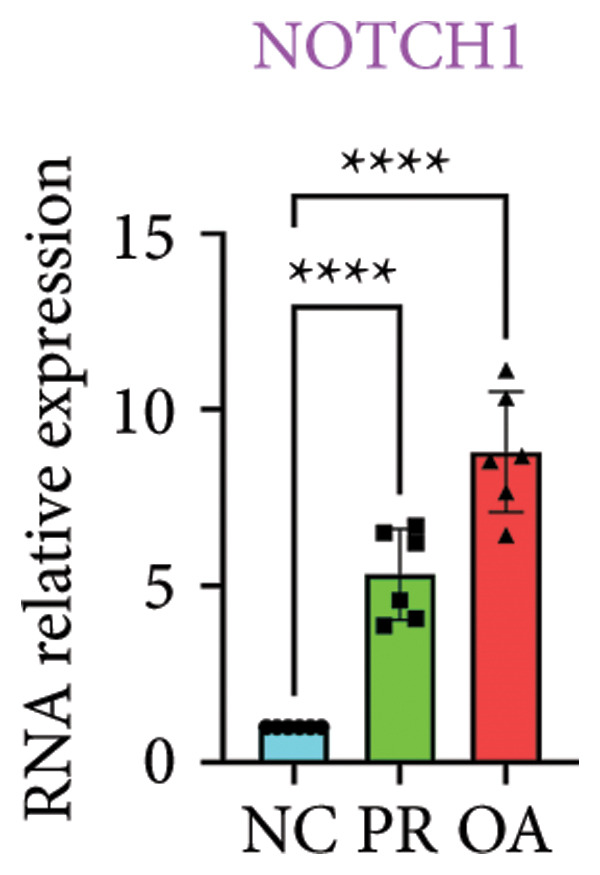
(p)

**Figure:**
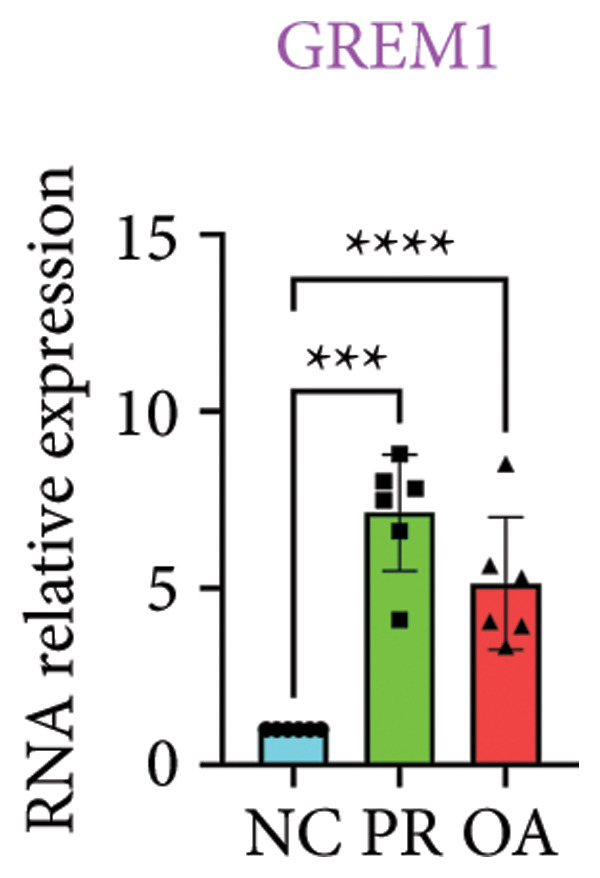
(q)

**Figure:**
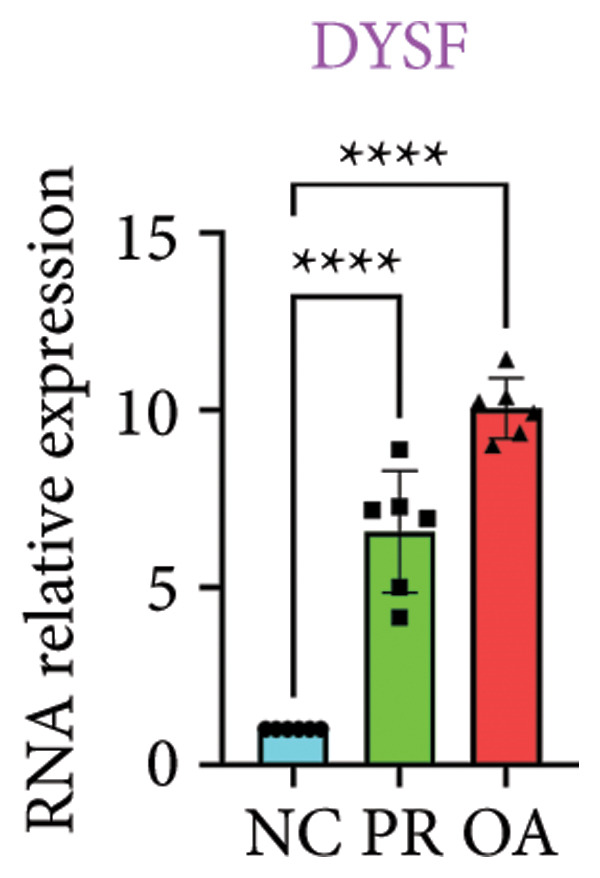
(r)

**Figure:**
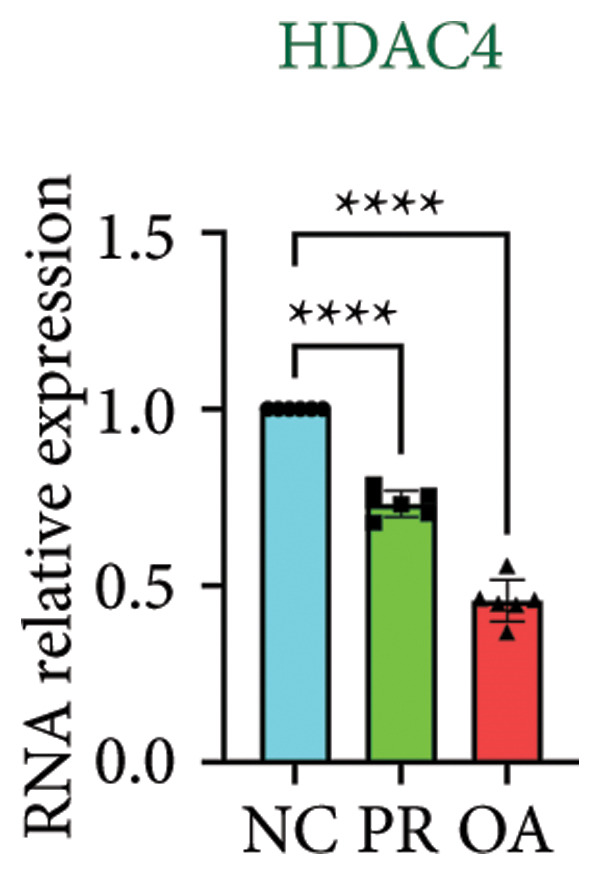
(s)

**Figure:**
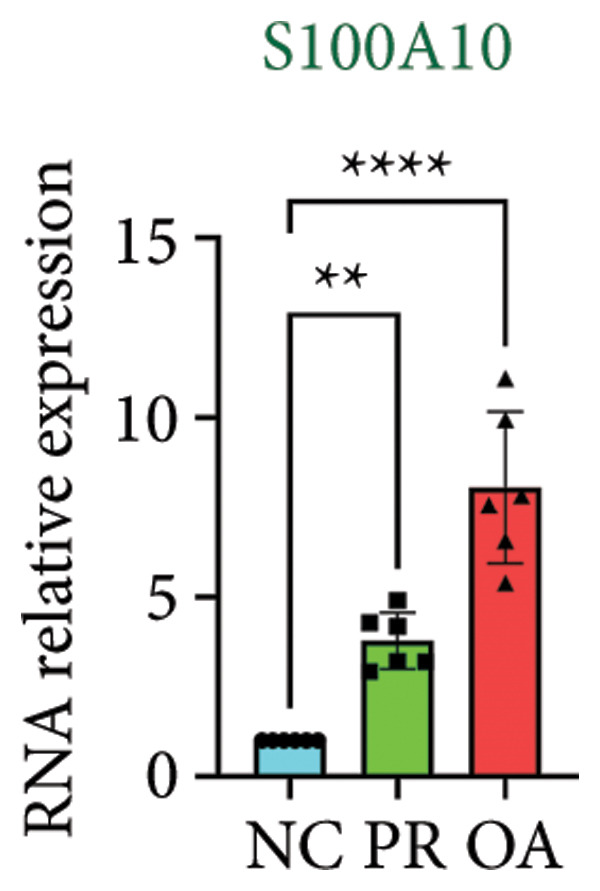
(t)

**Figure:**
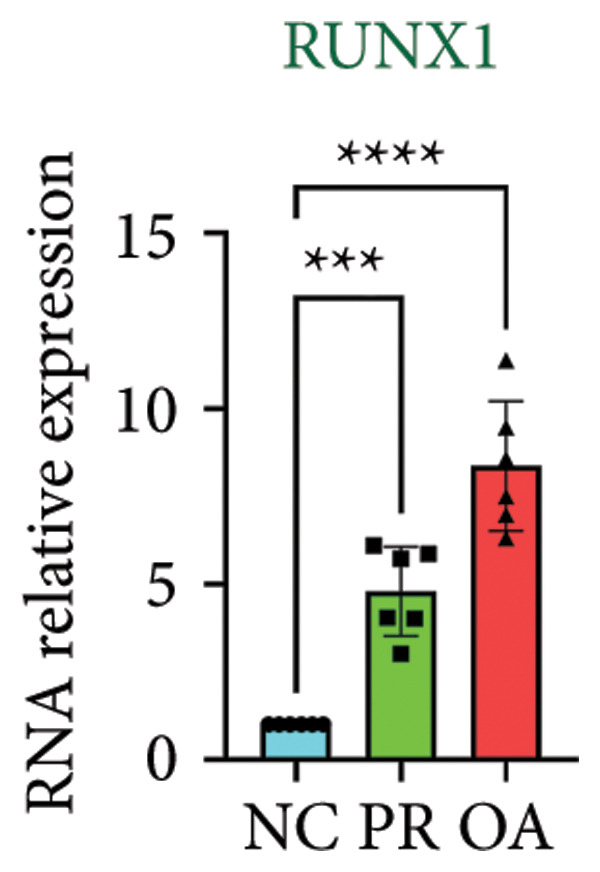
(u)

We collected hip cartilages from six patients of femoral neck fracture and six patients with hip OA who underwent total hip arthroplasty. We analyzed the ages of patients with femoral neck fractures and those with hip OA; there was no significant difference in age between the two groups (Supporting Figure 2A). Western blot analyses showed increased expression of NOTCH1, GREM1, and DYSF in hip OA cartilage (Figures 4(i), 4(j), 4(k), and 4(l)), decreased expression of HDAC4, and increased expression of S100A10 and RUNX1 in hip OA cartilage (Figures 4(i), 4(m), 4(n), and 4(o)). We then extracted RNA for validation. Relative expression of hypermethylated genes showed increased expression of NOTCH1, GREM1, and DYSF in hip OA cartilage (Figures 4(p), 4(q), and 4(r)), and relative expression of hypomethylated genes showed decreased expression of HDAC4 and increased expression of S100A10 and RUNX1 in hip OA cartilage (Figures 4(s), 4(t), and 4(u)). RNA expression of these genes was consistent with methylation position in gene region, like TSS1500 in S100A10 and RUNX1, and gene body in NOTCH1, DYSF, and HDAC4.

## 4. Discussion

Emerging studies have shown that epigenetics play an important role in the pathogenesis of OA, of which DNA methylation is the dominant [14, 16, 17, 20]. We described DNA methylation of the whole genome in hip cartilages during OA, which contained 19 cartilages of femoral neck fracture and 14 PRs and 30 damaged cartilages from OA patients. We found a lot of DMPs in preserved and damaged cartilages compared to control cartilages. Interestingly, there were no DMPs detected between PRs and damaged cartilages, despite the PR had a normal morphology, while the morphology of damaged cartilage was wearing. This was inconsistent with the published reports, which found 245 unique differentially methylated genes in knee and hip articular cartilages [18]. This might be because we employed a stricter screening criteria with deltaBeta value over 0.2 (different from 0.1 they used) in order to eliminate false positives as much as possible. Moreover, with more damaged cartilage samples, these differences disappeared. However, in our subsequent analyses, we identified that this discrepancy may indeed exist. This could potentially be attributed to the fact that the samples in the PR group and the OA group of the GEO dataset were not derived from the same patient, whereas the two groups we selected were indeed obtained from the same patient. Given the considerable heterogeneity among human tissue samples, this likely accounts for the observed difference. Our results indicated that the change of the microenvironment (whether the patient had clinical diagnostic symptoms of OA) was one of the important factors affecting the methylation of hip cartilage.

Studies had shown the comparison of DNA methylation between OA and non‐OA hip cartilages and the comparison of DNA methylation between preserved and damaged hip cartilages [14, 18]. However, we combined the two GEO datasets (GSE63106 and GSE63695) to identify the differential methylated genes across the whole genome not only on PR but also on damaged cartilage in OA, using cartilages of femoral neck fracture as the negative control. The identification of specific methylation alterations in OA may offer a novel approach to detect early OA. This was achieved by analyzing the corresponding circulating DNA fragments in patient blood or joint fluid, which act as biomarkers before structural changes are visible. Consequently, this method facilitates timely and precise treatment, thereby slowing the pathological process of OA [21]. The PI3K‐AKT and AMPK signaling pathways also play critical roles in maintaining chondrocyte homeostasis and suppressing inflammatory responses; dysregulation of these pathways is strongly implicated in the pathogenesis of OA. The study of chondrocyte methylation has enabled us to have a clearer understanding of OA. As far as we know, no current methylation profiling approach has simultaneously analyzed these three distinct cartilage states. Our study, which examines femoral neck fracture cartilage, intact cartilage, and damaged hip joint cartilage, provides valuable methylation data that may contribute to the early diagnosis and elucidation of the pathogenesis of early OA.

Among these top hypermethylated genes, several genes have been reported to be associated with OA in GWAS, such as DYSF, fibroblast growth factor receptor 3 (FGFR3), and collagen type XI alpha 2 chain (COL11A2) [6, 22, 23]. DYSF was a skeletal muscle protein found to be associated with the sarcolemma, and mutation in mice showed significant skeletal muscle necrosis with macrophage infiltration [24]. FGFR3 was strongly implicated in OA, and FGFR3 conditional knockout mice aggravated destabilization of the medial meniscus (DMM)‐induced cartilage degradation [25, 26]. Disruption of COL11A2 resulted in a mild cartilage phenotype, of which articular cartilage was thinner and cells within appeared to be disorganized [27]. While in hypomethylated genes, GLIS family zinc finger 3 (GLIS3) was reported to be associated with the susceptibility of OA [6, 7, 28, 29]. GLIS3 has been identified to be upregulated in the cartilage of OA patients, and studies showed cryptotanshinone‐protected cartilage through miR‐106a‐5p/GLIS3 axis in OA [30].

The expressions of three hypermethylated genes (NOTCH1, GREM1, and DYSF) and three hypomethylated genes (HDAC4, S100A10, and RUNX1) were evaluated in NC and preserved and damaged cartilages, which were in agreement with the methylation in gene body, 5′UTR, or TSS1500 of these genes. NOTCH1 was a single‐pass transmembrane receptor, and inhibition of NOTCH1 in mice enhanced chondrocyte hypertrophy and increased severity of OA [31]. GREM1 was known to inhibit bone morphogenetic proteins (BMPs) and TGFβ signaling pathways, and it played essential roles in OA development [32]. HDAC4 was shown to alter cartilage homeostasis in human OA, and knockdown of HDAC4 attenuated the expression of catabolic genes in chondrocytes [33]. S100A10 was a member of the S100 family, and the suppression of S100A10 in human chondrocytes showed decreased expression of inflammatory cytokines, which could be a potential target for OA treatment [34]. RUNX1 played a key role in the homeostasis of articular cartilage, and conditional Runx1 knockout aggravated the loss of cartilage in OA [35, 36].

The molecular network here revealed the possible protein–protein interactions of epigenetics in OA. Although many studies have revealed molecular networks in OA, most of them were based on transcriptome changes [37–39]. While our results were based on changes in DNA methylation, which might bring us a lot of new information, in the future, it may be more meaningful to further combine transcriptome changes and DNA methylation results.

Although we have revealed many changes in DNA methylation during OA, there were still some limitations in this study. Firstly, it is important to note the limitations associated with using cartilage from femoral neck fractures as a control. While donor age was consistent across groups, the fracture‐associated cartilage originated largely from an elderly population and may not be fully representative of healthy tissue. In subsequent work, the inclusion of normal cartilage from healthy donors will be essential to confirm and extend the conclusions of this study. Secondly, the number of control samples was limited, and more control samples or normal cartilages from heathy donors would make the results more meaningful. Thirdly, further molecular experiment was needed to demonstrate the functions of these genes or the methylation positions. There might be two or more DMPs which located in different gene regions, and it cannot be distinguished which one was dominant and affected gene expression. It would be very helpful to explain the changes of these methylation positions if combining transcriptomic or proteomic data in OA.

In conclusion, we identified methylation changes in preserved and damaged cartilages in OA and verified the expression of six methylated genes in human hip samples. We hope these findings can help to determine potential biomarkers or therapeutic targets for OA.

## Disclosure

All authors approved the final version of the manuscript.

## Conflicts of Interest

The authors declare no conflicts of interest.

## Author Contributions

D.S. conceived and supervised the study. M.W., G.T., and R.J. performed data analysis and wrote the manuscript. RW edited the manuscript. G.T., R.J., Y.L., and J.L. collected data. R.J. and M.W contributed equally to this work.

## Funding

This study was funded by the National Natural Science Foundation of China, 82325035, 82172481, and 32271409; Six Talent Peaks Project in Jiangsu Province, WSW‐079; and Innovation Project of National Orthopedics and Sports Medicine Rehabilitation Clinical Medical Research Center, 2021‐NCRC‐CXJJ‐ZH‐16.

## Supporting Information

Supporting Figure 1: Boxplot and heatmap of all the methylation profiles. (A) Boxplot of raw data. (B) Boxplot of the methylation data after normalization. (C) Heatmap of DMPs in NC_PR. (D) Heatmap of DMPs in NC_OA. Supporting Figure 2: Ages of NC and OA. (A) Comparison of ages between the NC and the OA. ^∗^Statistical significance. ^∗^ *p* < 0.05. ^∗∗^ *p* < 0.01. ^∗∗∗^ *p* < 0.001. ^∗∗∗∗^ *p* < 0.0001. ns represents there is no statistical significance. NC, patients with femoral neck fracture; OA, patients with hip OA. Supporting Table 1: Characteristics of all the patients. Supporting Table 2: Differential methylated positions in NC_PR and NC_OA. Supporting Table 3: Gene ontology and KEGG analysis in NC_PR and NC_OA. Supporting Table 4: The primer sequences of mRNA.

## Supporting information


**Supporting Information** Additional supporting information can be found online in the Supporting Information section.

## Data Availability

The datasets generated or analyzed during the current study are available in the GEO database (GSE63106 and GSE63695).
