# Supplementary figures and images for: Methylation Landscapes of Cartilage in Hip Osteoarthritis
Source: Genet Res (Camb). 2026 Jan 5;2026:5540232. doi: 10.1155/genr/5540232 (PMC12766398; doi:10.1155/genr/5540232)

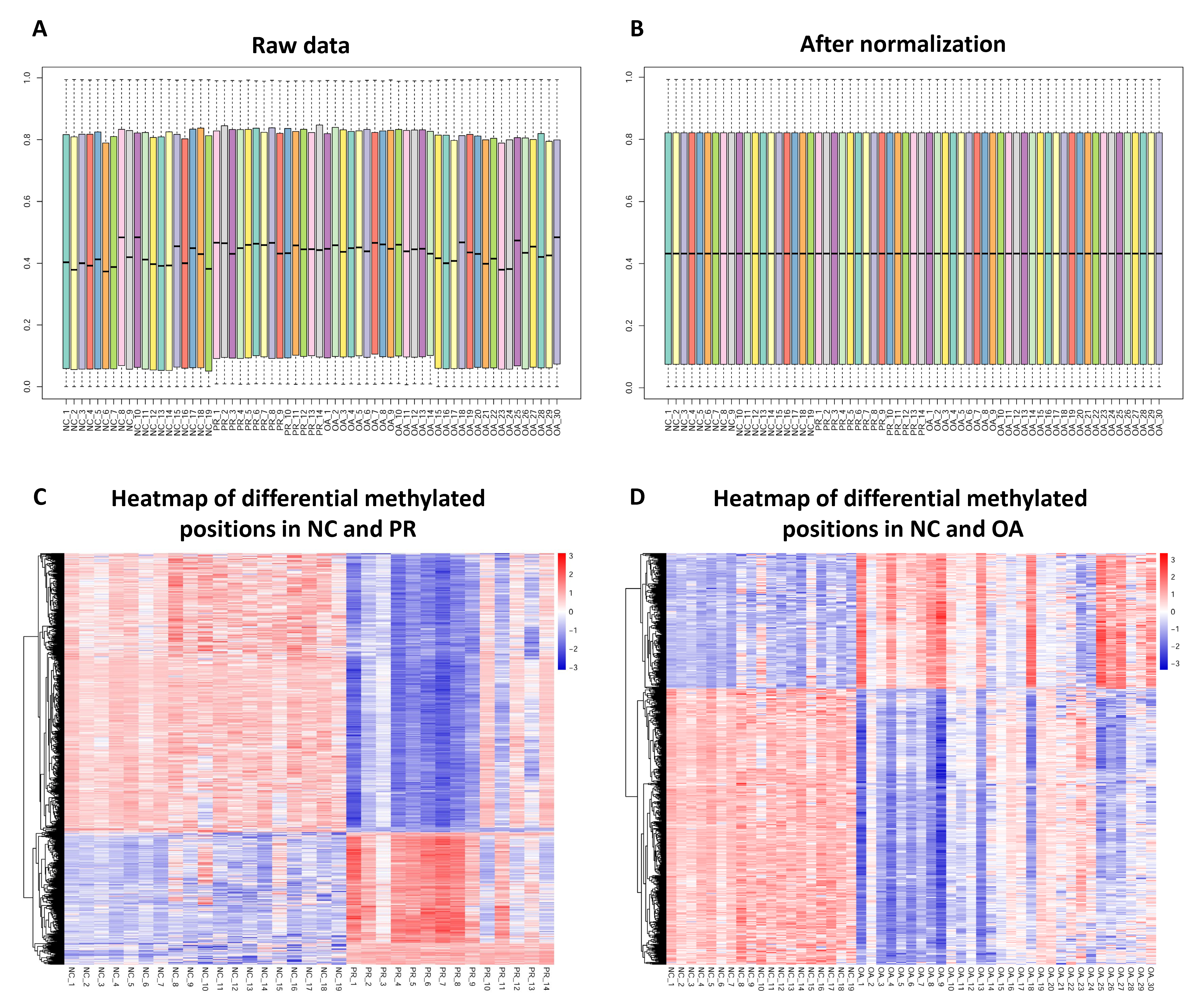

Supplement: Supplementary file 1 — Supporting Information Additional supporting information can be found online in the Supporting Information section. [file GENR-2026-5540232-s001.zip › Supplementary fig 1.tiff]

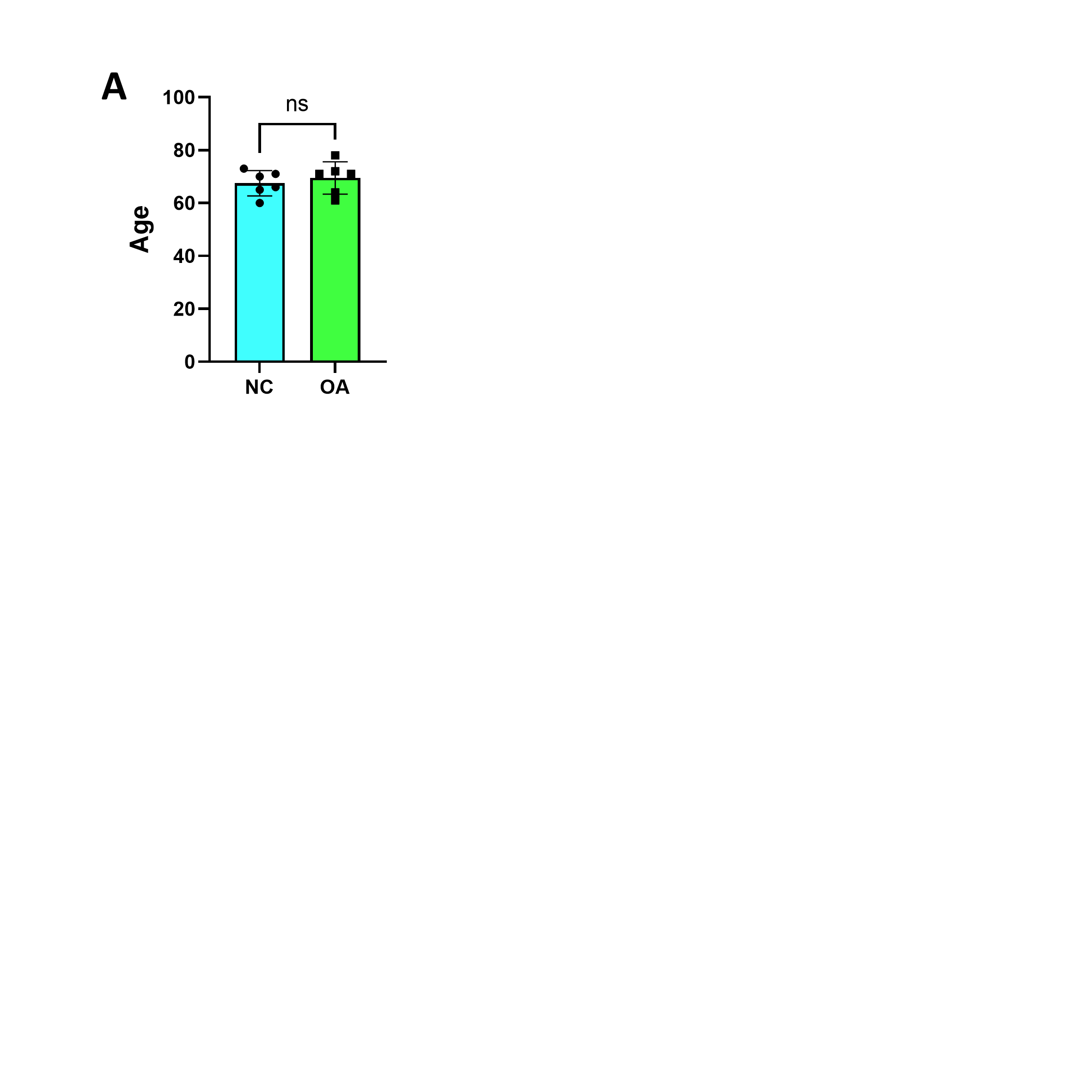

Supplement: Supplementary file 1 — Supporting Information Additional supporting information can be found online in the Supporting Information section. [file GENR-2026-5540232-s001.zip › supplementary fig2.tif]
